# Atomic layer deposition-based functionalization of materials for medical and environmental health applications

**DOI:** 10.1098/rsta.2010.0011

**Published:** 2010-04-28

**Authors:** Roger J. Narayan, Shashishekar P. Adiga, Michael J. Pellin, Larry A. Curtiss, Alexander J. Hryn, Shane Stafslien, Bret Chisholm, Chun-Che Shih, Chun-Ming Shih, Shing-Jong Lin, Yea-Yang Su, Chunming Jin, Junping Zhang, Nancy A. Monteiro-Riviere, Jeffrey W. Elam

**Affiliations:** 1 Joint Department of Biomedical Engineering, University of North Carolina and North Carolina State University, 2147 Burlington Engineering Labs, Raleigh, NC 27695-7115, USA; 2 Kodak Research Laboratories, Eastman Kodak Company, Rochester, NY 14650, USA; 3 Materials Science Division, Argonne National Laboratory, Argonne, IL 60439, USA; 4 Energy Science Division, Argonne National Laboratory, Argonne, IL 60439, USA; 5 Center for Nanoscale Science and Engineering, North Dakota State University, 1805 Research Park Drive, Fargo, ND 58102, USA; 6 Institute of Clinical Medicine,National Yang-Ming University, Taipei 112, Taiwan, Republic of China; 7 Cardiovascular Research Center, National Yang-Ming University, Taipei 112, Taiwan, Republic of China; 8 Division of Cardiovascular Surgery, Taipei Veterans General Hospital, Taipei 112, Taiwan, Republic of China; 9 Division of Cardiology, Taipei Veterans General Hospital, Taipei 112, Taiwan, Republic of China; 10 Graduate Institute of Medical Sciences, School of Medicine, Taipei Medical University, Taipei 110, Taiwan, Republic of China; 11 Center for Chemical Toxicology Research and Pharmacokinetics, North Carolina State University, Raleigh, NC 27606, USA

**Keywords:** atomic layer deposition, self-assembly, nanoporous alumina, antimicrobial, antifouling

## Abstract

Nanoporous alumina membranes exhibit high pore densities, well-controlled and uniform pore sizes, as well as straight pores. Owing to these unusual properties, nanoporous alumina membranes are currently being considered for use in implantable sensor membranes and water purification membranes. Atomic layer deposition is a thin-film growth process that may be used to modify the pore size in a nanoporous alumina membrane while retaining a narrow pore distribution. In addition, films deposited by means of atomic layer deposition may impart improved biological functionality to nanoporous alumina membranes. In this study, zinc oxide coatings and platinum coatings were deposited on nanoporous alumina membranes by means of atomic layer deposition. PEGylated nanoporous alumina membranes were prepared by self-assembly of 1-mercaptoundec-11-yl hexa(ethylene glycol) on platinum-coated nanoporous alumina membranes. The pores of the PEGylated nanoporous alumina membranes remained free of fouling after exposure to human platelet-rich plasma; protein adsorption, fibrin networks and platelet aggregation were not observed on the coated membrane surface. Zinc oxide-coated nanoporous alumina membranes demonstrated activity against two waterborne pathogens, *Escherichia coli* and *Staphylococcus aureus*. The results of this work indicate that nanoporous alumina membranes may be modified using atomic layer deposition for use in a variety of medical and environmental health applications.

## 1. Introduction

There is a great interest in incorporating nanoporous materials within devices for medical and environmental health applications. As defined by Lu & Zhao (2004), nanoporous materials contain pore diameters between 1 and 100 nm and large porosities (volume ratio of pore space to total material volume greater than 0.4). According to Rouquerol *et al.* (1994), nanoporous materials may be classified by pore diameter as microporous (pore diameters smaller than 2 nm), mesoporous (diameters between 2 and 50 nm) or macroporous (pore diameters larger than 50 nm). Analogous structures to nanoporous materials can be found in many biological organisms. An example of a natural filter is the nanostructured epithelial cell in the kidney, which is known as the podocyte. The podocyte allows water and small waste molecules to pass into the urine. On the other hand, it pre- vents proteins (e.g. albumin) and cells from passing into the urine. Karnovsky & Ainsworth (1972) described glomerular filtration in the kidney as a two-part process, in which the glomerular basement membrane serves as a coarse filter and the slit diaphragm serves as a molecular sieve. Rodewald & Karnovsky (1974) subsequently demonstrated, using electron microscopy, that the slit diaphragm contains rectangular pores; the dimensions of the filtration slit are 4×14 nm.

Recent studies by Huang *et al.* (2007) and Narayan *et al.* (2007) have examined the use of anodized aluminium oxide, also known as nanoporous alumina, as a membrane for kidney dialysis (a process that replaces natural kidney function) owing to its high porosity, uniform pore size and stability at high temperatures. The anodization process allows for the development of a semipermeable membrane with ideal properties for controlled filtration of nanoscale materials. Unlike many porous materials, the pores in nanoporous alumina exhibit a narrow distribution of pore sizes as well as well-defined pore geometries. In addition, Uehara *et al.* (2009) noted that nanoporous alumina membranes are unusual among nanoporous materials with regard to the straightness of the pores over thicknesses exceeding 1 μm.

Several investigators have examined the use of anodization for fabrication of nanoporous alumina membranes. Raj *et al.* (2003) noted that anodizing is a common method for forming an oxide coating on aluminium and its alloys in order to inhibit corrosion. Keller *et al.* (1953) used electron microscopy to demonstrate that anodizing aluminium in acid electrolytes results in a thick layer of nearly cylindrical pores, which are oriented perpendicularly to the surface of the material. The regular, nearly cylindrical pores are arranged in a close-packed hexagonal cell structure. O’ Sullivan & Wood (1970) subsequently demonstrated that pore formation occurs as a result of concentration of current into residual thin areas of the anodic oxide surface. In their study, cell diameter and pore diameter were shown to be directly related to anodizing voltage; a proportionality factor of 2.8 nm V^−1^ was suggested. According to Darder *et al.* (2006), thickness is related to the amount of transferred charge. Work by Jessensky *et al.* (1998) indicated that the relationship between pore dimensions and anodizing voltage can be correlated with the current efficiency for oxide formation as well as with the volume expansion of aluminium during oxide formation. Garcia-Vergarai *et al.* (2006) stated that alumina–electrolyte chemical interactions are also affected by the composition of the electrolyte, the pH and the processing temperature. Jessensky *et al.* (1998) suggested that self-organization of nanoscale pores results from mechanical stress owing to the volume expansion of aluminium; repulsive forces between pores provide the driving force for self-organization. Nielsch *et al.* (2002) determined that the volume expansion of alumina to aluminium is approximately 1.2. Su *et al.* (2008) created an equifield strength model to explain pore formation and self-adjustment of pore ordering in nanoporous alumina. The relative dissociation rate of water was shown to play a significant role in determining the ratio of pore size to cell dimension.

Contemporary interest in the use of nanoporous alumina as a template for the development of nanostructured materials and devices can be traced back to the work by Masuda & Fukuda (1995). Highly ordered platinum and gold nanohole arrays were prepared using a replication process, which involved initial formation of a negative structure of nanoporous alumina and subsequent formation of a positive structure of a nanoporous metal. These structures exhibited 70 nm dia- meter pores, 1–3 mm thickness and textured surfaces. Masuda *et al.* (1997) subse- quently demonstrated the fabrication of nanoporous alumina using a textured surface pattern; arrays with aspect ratios greater than 150 and channel densities of 10×10^−10^ cm^−2^ were demonstrated. The textured surface pattern was prepared by means of a reusable mould. Li *et al.* (1999) described a two-step anodization process to fabricate arrays with 6×10^8^ to 5×10^10^ cm^−2^ pore densities; as with the single-step process described earlier, the interpore distance was altered by modifying the anodizing voltage as well as by modifying the anodic electrolyte. Recent efforts have involved fabrication of nanoporous alumina using hard anodi- zation processes. For example, Lee *et al.* (2006) used a hard anodization process in order to prepare nanoporous alumina membranes; membranes with uniform pores, high aspect ratios (greater than 1000) and pore diameters between 40 and 60 nm were created using this process. Vojkuvka *et al.* (2008) also prepared nanoporous alumina by means of a hard anodization process; membranes with long interpore distances, uniform pore sizes and good pore ordering were prepared.

Owing to its chemical stability, large surface area, high pore density, well-controlled and uniform pore size, as well as straight pores, nanoporous alumina has been investigated for use in several medical and environmental applications over the past decade. Nanoporous alumina membranes can be processed with smaller pore sizes and more uniform pore sizes than polymer membranes. Unlike other inorganic materials such as silicon, alumina is stable in physiological solutions and does not induce calcium phosphate deposition (Bayliss *et al.* 1999; Anglin *et al.* 2008). For example, Gong *et al.* (2003) demonstrated diffusion of dextran conjugates and fluorescein isothiocyanate from nanoporous alumina capsules with pore sizes between 25 and 55 nm. Molecular transport was controlled by selecting a nanoporous alumina capsule with an appropriate capsule pore size. Nanoporous alumina capsules were shown to prevent diffusion of molecules larger than a given cut-off size. They were able to create membranes with branched pores that exhibited less than 10 nm pore sizes using multiple anodizing voltages; the small pores were supported by, and were connected to, larger pores. Orosz *et al.* (2004) proposed the use of nanoporous alumina membranes for delivery of angiostatic and antioxidant pharmacological agents. Diffusion of catalase, endostatin and vitamin C through nanoporous alumina membranes for modulation of human retinal endothelial cell activity was demonstrated. It is believed that nanoporous materials such as nanoporous alumina may enable quasi-linear release kinetics and may provide slower transport rates than conventional sustained release devices. Kipke & Schmid (2004) demonstrated the diffusion of crystal violet-containing micelles encapsulated in sodium dodecylsulphate through nanoporous alumina membranes with pore sizes between 20 and 200 nm; the release of free crystal violet into an aqueous environment was spectroscopically demonstrated. Swan *et al.* (2005*a*) physically adsorbed vitronectin and covalently immobilized arginine–glycine–aspartic acid–cysteine cellular adhesive peptide onto nanoporous alumina surfaces in order to enhance the adhesion of osteoblasts. Arginine–glycine–aspartic acid–cysteine cellular adhesive peptide immobilization was shown to improve osteoblast adhesion; matrix production was demonstrated on the peptide-coated surfaces. Swan *et al.* (2005*b*) also showed that osteoblasts grown on nanoporous alumina (pore diameter=30–80 nm) extended cell processes into the pores; the cells exhibited normal phenotype and morphology. Popat *et al.* (2005) seeded osteoblasts on nanoporous alumina membranes, aluminium as well as other materials; short-term osteoblast adhesion and proliferation were shown to be better on nanoporous alumina membranes than on other surfaces. Osteoblasts on nanoporous alumina membranes deposited more matrix and showed higher protein content than cells on other surfaces. Darder *et al.* (2006) developed an amperometric biosensor, in which glucose oxidase was encapsulated by a nanoporous alumina membrane. The large surface area of the nanoporous alumina membrane allowed relatively large amounts of glucose oxidase to be incorporated. Yang *et al.* (2007) immobilized urease on nanoporous alumina membranes; the immobilized enzyme served as the basis of an electrode-separated piezoelectric sensor. The urea sensor exhibited short response times, high selectivity for urea as well as long-term storage stability. La Flamme *et al.* (2007) examined the use of nanoporous alumina and poly(ethylene glycol)-modified nanoporous alumina as membranes for immunoisolation devices. The surfaces of nanoporous alumina membranes were modified with poly(ethylene glycol) in order to resist the adhesion of blood proteins as well as immune cells; proteins and cells may block pores and reduce diffusion across an immunoisolation device membrane. Nanoporous alumina and poly(ethylene glycol)-modified nanoporous alumina were shown to be non-toxic and did not initiate significant complement activation. In addition, poly(ethylene glycol)-modified nanoporous alumina was shown to exhibit fewer interactions with serum albumin than unmodified nanoporous alumina. Poly(ethylene glycol)-modified nanoporous alumina was also shown to minimize the host response when implanted within the peritoneal cavity of rats. Attaluri *et al.* (2009) compared the transport performance of nanoporous alumina membranes with polyethersulphone membranes used in haemodialysis; the hydraulic conductivity of nanoporous alumina was shown to be twice that of polyethersulphone. In addition, no albumin leakage was noted from albumin membranes. Parkinson *et al.* (2009) utilized a nanoporous alumina template in order to create nanoporous membranes for fibroblast growth. Dermal fibroblasts and epidermal keratinocytes were shown to adhere to these nanoporous membranes as well as migrate across membrane surfaces. Graham *et al.* (2009) recently suggested that nanoporous alumina electrodes may be used as an interface with neuronal cells; an *in vitro* assay involving mouse neuroblastoma × rat glioma hybrid cells demonstrated the biocompatibility of nanoporous alumina. The use of nanoporous alumina membranes for environmental applications has also received recent interest. For example, Ma, B. *et al.* (2009) suggested the use of nanoporous membranes for water purification.

As mentioned earlier, many medical and environmental applications for nanoporous alumina membranes are attributed to the fact that these materials exhibit high pore densities, well-controlled and uniform pore sizes, as well as straight pores. Transport of materials across nanoporous membranes may be controlled by altering the membrane pore size in a controlled manner. In addition, nanoporous materials with unusual biological and chemical properties may be created by chemically modifying the pore surface. For example, Xiong *et al.* (2005) pointed out that impurities incorporated within nanoporous alumina membranes during the synthesis of these structures could have a significant influence on the chemical properties of the membranes. In addition, it is currently unknown whether aluminium is a biocompatible material; for example, Hegde *et al.* (2003) suggested that aluminium ingestion is associated with the development of amyloid plaques. Atomic layer deposition may be used to decrease the sizes of the pores while retaining a narrow pore distribution. In addition, the coatings may impart improved biological and chemical properties to the nanoporous alumina membranes. Atomic layer deposition is a thin-film growth process that involves alternating chemical reactions between gaseous precursor molecules on the surface of a material. According to Puurunen & Vandervorst (2004), the self-terminating gas–solid reactions enable deposition of atoms or molecules in a layer-by-layer fashion. In atomic layer deposition, individual reactions are separated by purge steps involving saturation with an inert gas. By saturating the substrate during each reaction, all of the surfaces of a substrate receive a conformal coating of identical thickness. As such, atomic layer deposition is uniquely suited for depositing conformal nanometre-scale films with precise thickness values onto nanoporous membrane surfaces. Stair *et al.* (2006) described the fabrication of highly uniform heterogeneous catalysts by atomic layer deposition of vanadium oxide coatings on nanoporous alumina membranes. Vanadium oxide-coated nanoporous alumina membranes were shown to provide improved selectivity for catalytic oxidative dehydrogenation of cyclohexane compared with conventional powdered alumina-supported vanadia catalysts. Moon *et al.* (2009) used atomic layer deposition to reduce the pore size in nanoporous alumina membranes from 70 to 40 nm. Centrifugation was subsequently used to align bacteriophage phi29 virus nanoparticles in the pores of the nanoporous alumina membranes. Selective filtration of nanoparticles was obtained; empty capsids entered the 20 nm pores and DNA-packaged particles remained on the membrane surface. Velleman *et al.* (2009) demonstrated surface modification of nanoporous alumina membranes using silica; these silica-coated membranes were subsequently modified with perfluorodecyldimethylchlorosilane. These hydrophobic membranes showed enhanced transport of hydrophobic molecules such as (tris(2,2′-bipyridyl)dichlororuthenium(II) hexahydrate) over hydrophilic molecules such as Rose Bengal. Adiga *et al.* (2008) described the use of atomic layer deposition to modify the surfaces of membranes; these materials have applications in size-sorting and filtration of small biological molecules. They showed that a PEGylated, platinum-coated nanoporous alumina membrane exposed to human platelet-rich plasma resisted fouling by proteins and platelets. Narayan *et al.* (2009) deposited titanium oxide coatings onto nanoporous alumina membranes with pore sizes of 20 and 100 nm by means of atomic layer deposition. Neither the titanium oxide-coated 20 nm nanoporous alumina membranes nor the titanium oxide-coated 100 nm nanoporous alumina membranes exhibited statistically lower viability than the uncoated nanoporous alumina membrane control materials. In addition, titanium oxide-coated 20 nm nanoporous alumina membranes exposed to ultraviolet light exhibited antimicrobial activity against two micro-organisms, *Escherichia coli* and *Staphylococcus aureus*. More recently, Lee *et al.* (2009) described the processing of photocatalytic zinc oxide and titania coatings on avian eggshell using atomic layer deposition.

This paper examines the use of atomic layer deposition for coating all of the surfaces of commercially obtained nanoporous alumina membranes, including the surfaces within the pores, with platinum or zinc oxide. In the first section, 20 nm pore size nanoporous alumina membranes were initially coated with platinum using atomic layer and subsequently coated with 1-mercaptoundec-11-yl hexa(ethylene glycol) using a self-assembly process; these membranes may be suitable for use in implantable biosensors. Human epithelial keratinocyte viability and human platelet-rich plasma–membrane interactions were examined. In the second section, 100 nm pore size nanoporous alumina membranes were conformally coated with zinc oxide using atomic layer deposition; these membranes have potential applications in water purification. Human epithelial keratinocyte viability and microbial growth on the zinc oxide-coated nanoporous alumina membranes were examined. Our results indicate that atomic layer deposition may be used to modify nanoporous alumina membranes as well as other nanostructured materials for medical and environmental applications.

## 2. Experimental procedure

The nanoporous alumina membranes were coated in a custom viscous flow atomic layer deposition reactor (inside diameter=5 cm), which was constructed from a circular stainless steel flow tube. A custom metal wire mesh fixture was used to vertically suspend the nanoporous alumina membranes in order to facilitate efficient diffusion of the gaseous precursors into the pores of the nanoporous alumina membranes; the fixture allowed up to 12 membranes to be coated at one time. Ultrahigh purity (99.999%) nitrogen carrier gas was continuously passed through the flow tube at a mass flow rate of 360 s.c.c.m. and at a pressure of 1 Torr. A constant reactor temperature was maintained by temperature controllers, which were connected to resistive heaters attached to the exterior of the reactor. Four separate heating zones were used to establish a uniform temperature profile along the length of the flow tube.

Platinum coatings were deposited on 20 nm pore size nanoporous alumina membranes using atomic layer deposition. The nanoporous alumina membranes were obtained from a commercial source (Whatman, Maidstone, UK). These membranes exhibited outside diameters of 13 mm and thicknesses of 60 μm. The nanoporous alumina membranes exhibited pore diameters of 200 nm for approximately 58 μm of the membrane thickness and tapered to 20 nm pore diameters for approximately 2 μm of the membrane thickness. Prior to coating, the nanoporous alumina membranes were cleaned *in situ* using a 5 min exposure to flowing ozone. The ozone was produced by an L11 commercial generator (Ozone Engineering, El Sobrante, USA). A temperature of 300^°^C and an ozone partial pressure of approximately 0.1 Torr were maintained during the cleaning process. The nanoporous alumina membranes were initially coated with alumina and subsequently coated with platinum. This alumina coating served to bury anionic impurities in the nanoporous alumina membranes. In addition, the alumina coating provided a densely hydroxylated surface in order to promote better nucleation of the platinum coating. Atomic layer deposition of the approximately 1 nm alumina coating was performed by means of alternating exposure to 10 cycles of 97 per cent purity trimethyl aluminium (Sigma-Aldrich, St Louis, USA) and water.

Precursor exposure times of 6 s were utilized for trimethyl aluminium and water; 5 s purge periods were used between precursor exposures to prevent mixing of trimethyl aluminium and water. The platinum coating was deposited using alternating exposures to 99 per cent purity trimethyl (methylcyclopentadienyl) platinum(IV) (Pt(MeCp)Me_3_) (Sigma Aldrich) and oxygen at a growth temperature of 300^°^C. Precursor exposure times of 50 and 20 s were utilized for Pt(MeCp)Me_3_ and oxygen, respectively; 10 s purge periods were used between precursor exposures to prevent mixing of Pt(MeCp)Me_3_ and oxygen. Silicon wafers were coated at the same time as the nanoporous alumina membranes. The thicknesses of the platinum and alumina coatings on the silicon wafers were determined using an M2000V spectroscopic ellipsometer (J. A. Woollam, Lincoln, USA). Imaging of the platinum-coated nanoporous alumina membranes was performed using an S4700 scanning electron microscope (Hitachi, Tokyo, Japan) with a field emission source as well as a 3200 scanning electron microscope (Hitachi) with a thermionic source and an Isis energy-dispersive X-ray spectrometer attachment (Oxford Instruments, Abingdon, UK).

A method similar to the one described by Wang *et al.* (2005) was used to obtain self-assembly of 1-mercaptoundec-11-yl hexa(ethylene glycol) monolayers on the platinum-coated nanoporous alumina membranes. 1-Mercaptoundec-11-yl hexa(ethylene glycol) (HS(CH_2_)_11_(OCH_2_CH_2_)_6_OH) and 99.5 per cent purity ethanol were obtained from a commercial source (Sigma Aldrich). Self-assembled monolayers were prepared by immersing platinum-coated nanoporous alumina membranes in an ethanol solution of 1-mercaptoundec-11-yl hexa(ethylene glycol) for 48 h. The membranes were subsequently removed from the solution, rinsed in ethanol, dried, sonicated in ethanol for 3 min, rinsed in ethanol and dried. Self-assembly of 1-mercaptoundec-11-yl hexa(ethylene glycol) monolayers was also performed on a silicon wafer that was coated with a titanium interlayer and a platinum top layer using pulsed laser deposition. Attenuated total reflectance Fourier transform infrared spectroscopy measurements were obtained from the coated silicon wafer using a Nicolet Nexus 470 Fourier transform infrared spectrometer equipped with an OMNI sampler and a continuum microscope (Thermo Fisher, Waltham, USA).

We examined the proliferation of neonatal human epidermal keratinocytes on PEGylated, platinum-coated nanoporous alumina membranes, platinum-coated nanoporous alumina membranes and uncoated nanoporous alumina membranes using the MTT (3-(4,5-dimethylthiazol-2-yl)2,5-diphenyl tetrazolium bromide) assay. The MTT assay, first described by Mossman (1983), is based on the reduction of a yellow tetrazolium salt (MTT) to a purple formazan dye by mitochondrial succinic dehydrogenase. Membranes were sterilized using ultraviolet B light, exposed for 3 h on each side, and rotated 90^°^ every 45 min. Upon completion of ultraviolet B light exposure, the membranes were placed in 24-well plates. A small drop of Akwa Tears (Akorn, Lake Forest, USA) was placed on the bottom of each well to secure the membranes and to prevent floating of the membranes. Wells and membranes were rinsed with 1 ml of keratinocyte growth medium (KGM-2) and were seeded with 20 000 human epidermal keratinocytes in 1 ml of KGM-2 per well. Medium was changed after 24 h. Once the human epidermal keratinocytes were 60 per cent confluent, they were grown for 24 h; the membranes were subsequently moved to new plates in order to prevent cell growth outside the membranes from influencing the data. Each coating was run in triplicate. Viability data for the PEGylated, platinum-coated membranes and platinum-coated membranes were standardized to the data for the uncoated nanoporous alumina membranes.

Platelet adhesion testing was used for evaluating adsorption of platelets, proteins and other blood components to the surfaces of PEGylated, platinum-coated nanoporous alumina membranes, platinum-coated nanoporous alumina membranes and uncoated nanoporous alumina membranes; Narayan *et al.* (2006) previously provided details on the platelet adhesion testing procedure. Fresh whole blood was obtained from a healthy human adult volunteer. The blood was examined for the presence of anticoagulants, pharmacological agents or other agents that could affect protein–biomaterial or platelet–biomaterial interaction by means of platelet function, prothrombin time and partial thromboplastic time studies. Sodium citrate was added to the blood in order to prevent coagulation. The blood was subsequently centrifuged using a Plasma Saver system (Haemonetics, Braintree, USA) at 25^°^C for 10 min, 25^°^C for 10 min and 4^°^C for 1 h (operating speed=3500 r.p.m.). According to Lozada *et al.* (2001), the first spin process in this multi-step centrifugation process separated low-platelet concentrated plasma from red blood cells and platelet-rich plasma. The mixture of platelet-rich plasma and red blood cells was separated; the platelet-rich plasma was subsequently collected at the bottom of the test tube owing to its high specific gravity. The platelet-rich plasma was incubated at 37^°^C for 10 min and subsequently frozen until testing. PEGylated, platinum-coated nanoporous alumina membranes, platinum-coated nanoporous alumina membranes and uncoated nanoporous alumina membranes were immersed in the platelet-rich plasma solution and incubated at 37^°^C for 10 min. Weakly adherent platelets were removed by rinsing the membranes with 0.9 per cent saline solution. The platelet-rich plasma-exposed membranes were subsequently fixed in 4 per cent glutaraldehyde and critical point-dried. Adhesion of proteins and platelets on platelet-rich plasma-exposed membranes was examined using a 3200 scanning electron microscope (Hitachi, Tokyo, Japan) with an Isis energy-dispersive X-ray spectrometer attachment (Oxford Instruments, Abingdon, UK).

Zinc oxide coatings were deposited on 100 nm pore size nanoporous alumina membranes using atomic layer deposition. The nanoporous alumina membranes were obtained from a commercial source (Whatman). These membranes exhibited outside diameters of 13 mm and thicknesses of 60 μm. The nanoporous alumina membranes exhibited pore diameters of 200 nm for approximately 58 μm of the membrane thickness and tapered to pore diameters of 100 nm for approximately 2 μm of the membrane thickness. Prior to coating, the nanoporous alumina membranes were cleaned *in situ* using a 5 min exposure to flowing ozone. A feed of ultrahigh purity oxygen at a flow rate of 400 s.c.c.m. was used to produce an ozone partial pressure of approximately 0.1 Torr. Atomic layer deposition of zinc oxide was performed by means of alternating exposure to diethyl zinc (Sigma Aldrich) and water at a temperature of 200^°^C. Precursor exposure times of 6 s were utilized for diethyl zinc and water. Partial pressures of diethyl zinc and water were maintained at approximately 0.2 Torr; 5 s purge periods were used between precursor exposures to prevent mixing of diethyl zinc and water. Thirty-one atomic layer deposition cycles were performed, which yielded a zinc oxide thickness of 5 nm. Silicon wafers were coated at the same time as the nanoporous alumina membranes. The thickness of the zinc oxide coating on the silicon wafer was determined using spectroscopic ellipsometry measurements. Imaging of the zinc oxide-coated nanoporous alumina membranes was performed using an S4700 scanning electron microscope (Hitachi, Tokyo, Japan) as well as a 3200 scanning electron microscope (Hitachi).

Powder X-ray diffraction measurements were performed on the zinc oxide-coated nanoporous membranes using a Miniflex Plus diffractometer (Rigaku, Tokyo, Japan). X-ray photoelectron spectroscopy was performed on the zinc oxide-coated nanoporous membranes using an LAS-3000 instrument (Riber, Bezons, France) with an Mg Kα anode source. We examined the proliferation of neonatal human epidermal keratinocytes on zinc oxide-coated nanoporous alumina membranes and uncoated nanoporous alumina membranes using the MTT assay. Membranes were sterilized and seeded as described above. Wells were seeded with 25 000 human epidermal keratinocytes in 1 ml of KGM-2 per well. Medium was changed after 24 h. Once the human epidermal keratinocytes were 60 per cent confluent, they were grown for 24 h; the membranes were subsequently moved to new plates in order to prevent cell growth outside the membranes from influencing the data. Each coating was run in triplicate. Viability data for the zinc oxide-coated nanoporous alumina membranes were standardized to the data for the uncoated nanoporous alumina membranes.

Microbial growth on zinc oxide-coated nanoporous alumina membranes and uncoated nanoporous alumina membranes was determined using the agar plating method. Liu *et al.* (2010) recently described the use of an agar plating assay to determine the antimicrobial properties of a novel water purification membrane; Copello *et al.* (2006) previously provided details on the agar plating procedure. Tryptic soy broth, tryptic soy agar, Luria–Bertani broth, Luria–Bertani agar, triphenyltetrazolium chloride and phosphate-buffered saline (×10) were purchased from a commercial source (VWR International, West Chester, USA). Phosphate-buffered saline (×1) was prepared in deionized water. Overnight cultures of *E. coli* ATCC 12435 (American Type Culture Collection, Manassas, USA) in Luria–Bertani broth and *S. aureus* ATCC 25923 (American Type Culture Collection) in tryptic soy broth were pelleted via centrifugation (4500 r.p.m.) for 10 min; the micro-organisms were subsequently resuspended in phosphate-buffered saline in order to obtain a final cell density of approximately 10^8^ cells ml^−1^. Sterile swabs were used to inoculate lawns of *E. coli* on Luria–Bertani agar plates and lawns of *S. aureus* on tryptic soy agar plates. Zinc oxide-coated nanoporous alumina membranes and uncoated nanoporous alumina membranes were then placed on the agar plates inoculated with *E. coli* or *S. aureus*. The plates were inverted and incubated for 24 h at 37^°^C in the dark. An additional set of zinc oxide-coated nanoporous alumina membranes and uncoated nanoporous alumina membranes was incubated under continuous exposure to a tungsten-halogen light source, which was placed in the incubator. Inhibition of bacterial growth on the membrane surfaces was evaluated visually from digital images, which were obtained after 24 h of incubation. A biological activity indicator dye, triphenyltetrazolium chloride, was added to the agar medium (70 mg l^−1^); this dye stained the micro-organisms a red colour, which aided in the visualization of microbial growth.

## 3. Results and discussion

**Figure 1.**
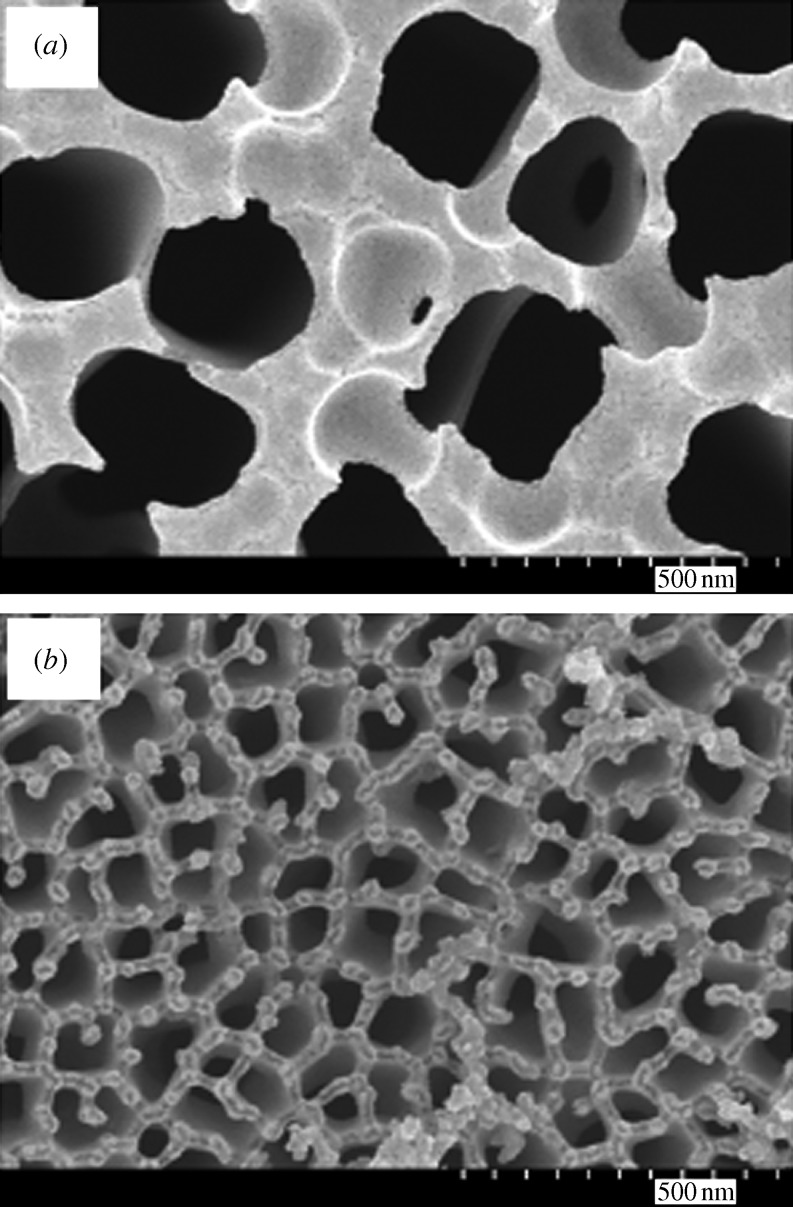
Plan-view scanning electron micrograph of a nanoporous alumina membrane following atomic layer deposition of 8 nm platinum coating. Images were obtained from (*a*) the large pore side of the membrane and (*b*) the small pore side of the membrane.

**Figure 2.**
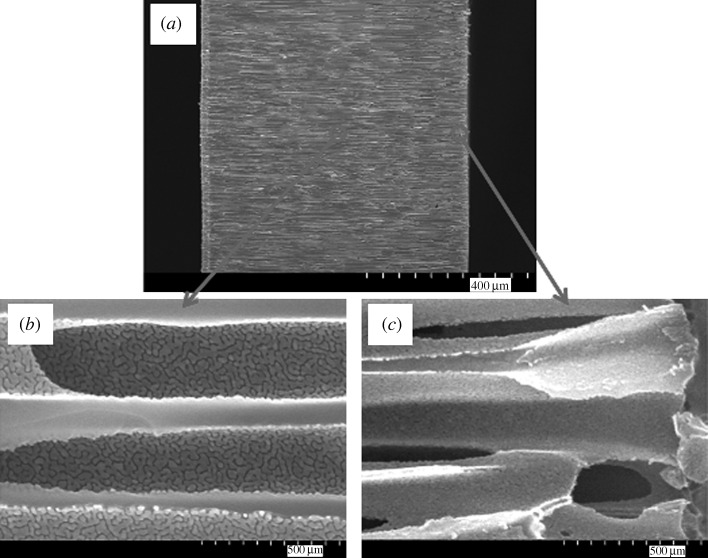
(*a*) Cross-sectional scanning electron micrograph obtained from a cleaved specimen of a nanoporous alumina membrane following atomic layer deposition of an 8 nm platinum coating. (*b*) High-resolution scanning electron micrograph at the middle of the pore shows a partially continuous platinum coating. (*c*) High-resolution scanning electron micrograph near the large pore edge shows a continuous platinum coating.

**Figure 3.**
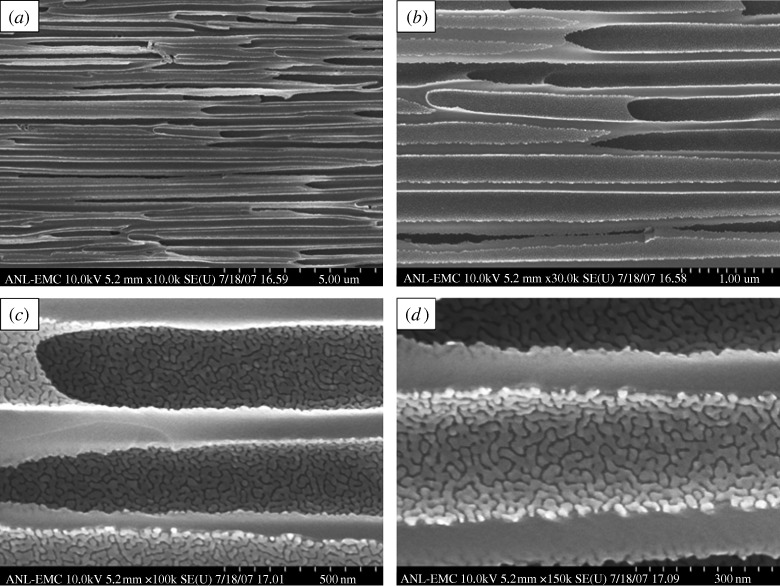
High-resolution scanning electron micrograph at the middle of the pore shows the island structure of the partially continuous platinum coating.

Figure 1*a* shows a plan-view scanning electron micrograph of a 20 nm pore size nanoporous alumina membrane following atomic layer deposition of an 8 nm platinum coating, which was obtained from the large pore side of the membrane. Figure 1*b* shows a plan-view scanning electron micrograph of a nanoporous alumina membrane following atomic layer deposition of an 8 nm platinum coating; the image was obtained from the small pore side of the membrane. These figures demonstrate that the nanoporous alumina membrane coated with platinum using atomic layer deposition exhibited a monodisperse pore size and high porosity. Figure 2 shows a cross-sectional scanning electron micrograph obtained from a cleaved 20 nm pore size nanoporous alumina membrane following atomic layer deposition of an 8 nm thick platinum coating. The coating is continuous near the ends of the pore (figure 2*b*); fairly uniform contrast was noted. Images obtained from near the small pore side of the platinum-coated nanoporous alumina membrane (data not shown) have a similar appearance. Figure 2*c* indicates that the platinum coating is partially continuous at the middle of the pore; uncoated regions of the nanoporous alumina membrane were observed. In order to evaluate the continuity of the platinum coating, electrical resistance through the nanoporous alumina membrane was determined using a digital ohmmeter; measurements were obtained by pressing the platinum-coated nanoporous alumina membrane between conductive metal plates. The resistance of the platinum-coated nanoporous alumina membranes was approximately 1Ω; in comparison, an immeasurably high resistance (greater than 20 MΩ) was obtained for the uncoated nanoporous alumina membranes. This result indicates that the platinum coating is partially continuous in nature. Figure 3 shows a high-resolution scanning electron micrograph obtained at the middle of the pore, which shows the island structure of the partially continuous platinum coating; platinum coatings prepared using atomic layer deposition typically consist of agglomerated platinum nanoparticles.

 Stair *et al.* (2006) demonstrated that atomic layer deposition is capable of producing highly uniform, conformal coatings on all exposed surfaces of nanoporous membranes. This attribute makes atomic layer deposition an attractive technology for not only chemically modifying the surfaces of nanoporous materials but also reducing pore size in nanoporous materials. As shown by Cameron *et al.* (2000), saturation of individual surface reactions in atomic layer deposition of metal oxide materials (e.g. titanium oxide) results in nearly ideal, layer-by-layer film growth. On the other hand, atomic layer deposition of noble metals such as platinum can sometimes result in the formation of partially continuous coatings consisting of isolated metal nanoparticles; this result is commonly obtained when thin platinum films are deposited on metal oxide surfaces. Partially continuous film growth results from a low density of nucleation sites for platinum on the metal oxide surface as well as from platinum sintering owing to surface diffusion. The partially continuous nature of the platinum coating has important implications for the biological functionality of platinum-coated nanoporous alumina membranes, since interactions between alumina and the surrounding environment may take place. A completely continuous platinum coating may be obtained using a larger number of cycles, using longer Pt(MeCp)Me_3_ exposures or using other mechanisms that increase film nucleation.

**Figure 4.**
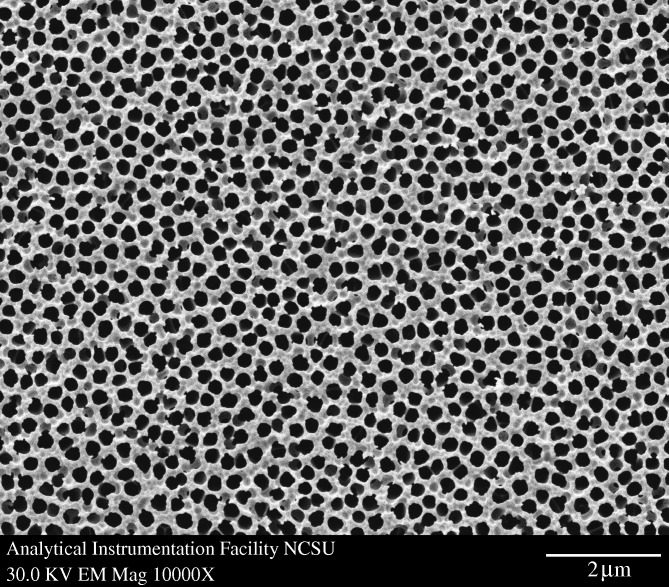
Plan-view scanning electron micrograph of a PEGylated, platinum-coated (coating=8 nm) 20 nm pore size nanoporous alumina membrane.

**Figure 5.**
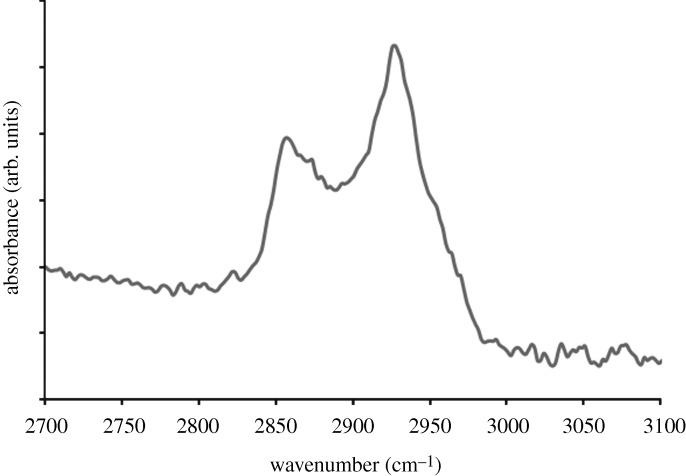
Fourier transform infrared spectrum of a PEGylated, platinum-coated, titanium-coated silicon wafer. Scale bar, 500 nm.

**Figure 6.**
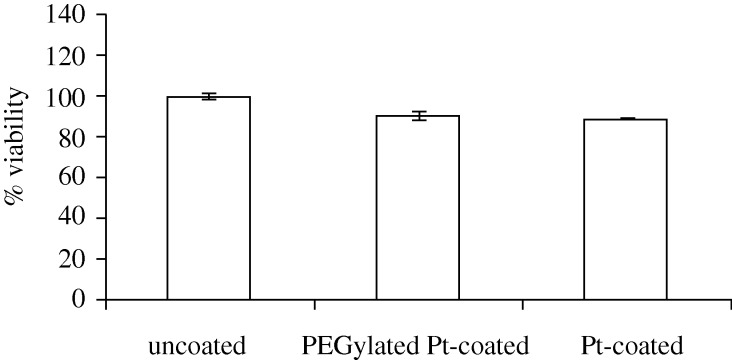
The 24 h MTT viability assay data for the PEGylated, platinum-coated (coating=8 nm) 20 nm pore size nanoporous alumina membrane, the platinum-coated (coating=8 nm) 20 nm pore size nanoporous alumina membrane and the uncoated 20 nm pore size nanoporous alumina membrane. Data were standardized to the uncoated membrane control. The PEGylated, platinum-coated membrane and the platinum-coated membrane demonstrated lower viability than the uncoated membrane.

Figure 4 shows a plan-view scanning electron micrograph of a platinum-coated (coating=8 nm) 20 nm pore size nanoporous alumina membrane after self-assembly of the 1-mercaptoundec-11-yl hexa(ethylene glycol) monolayer. No particulates, blockages or other alterations in pore morphology were observed on the surface of the PEGylated, platinum-coated nanoporous alumina membrane. The self-assembly process allowed the nanoscale pores on the surface of the platinum-coated membrane to be retained. The FTIR spectrum of a PEGylated, platinum-coated and titanium-coated silicon wafer is shown in figure 5; this spectrum showed good correspondence with the spectra of self-assembled monolayers containing oligo(ethylene glycol) moieties that were previously examined by Zolk *et al.* (2000) and Wang *et al.* (2005). Symmetric CH_2_ stretching vibrations were shown to extend over a range from 2850 to 2950 cm^−1^. The peak 2920 cm^−1^ was assigned to asymmetric CH_2_ stretching vibrations from the ethylene glycol chain. Figure 6 shows the 24 h MTT viability assay data for the PEGylated, platinum-coated (coating=8 nm) 20 nm pore size nanoporous alumina membrane, the platinum-coated (coating=8 nm) 20 nm pore size nanoporous alumina membrane and the uncoated 20 nm pore size nanoporous alumina membrane. The PEGylated, platinum-coated nanoporous alumina membrane and the platinum-coated nanoporous alumina membrane exhibited a decrease in viability compared with the uncoated nanoporous alumina membrane. Hexa(ethylene glycol) and platinum have demonstrated good cell compatibility in previous studies by Pang (1993) and Bajaj *et al.* (2007), respectively. Recent work by Narayan *et al.* (2008) suggested that the metal coatings formed galvanic couples with residual metallic aluminium in the nanoporous alumina membranes coated using a line-of-sight process known as pulsed laser deposition; these interactions accelerated aluminium ion release and led to reduced cell viability rates.

**Figure 7.**
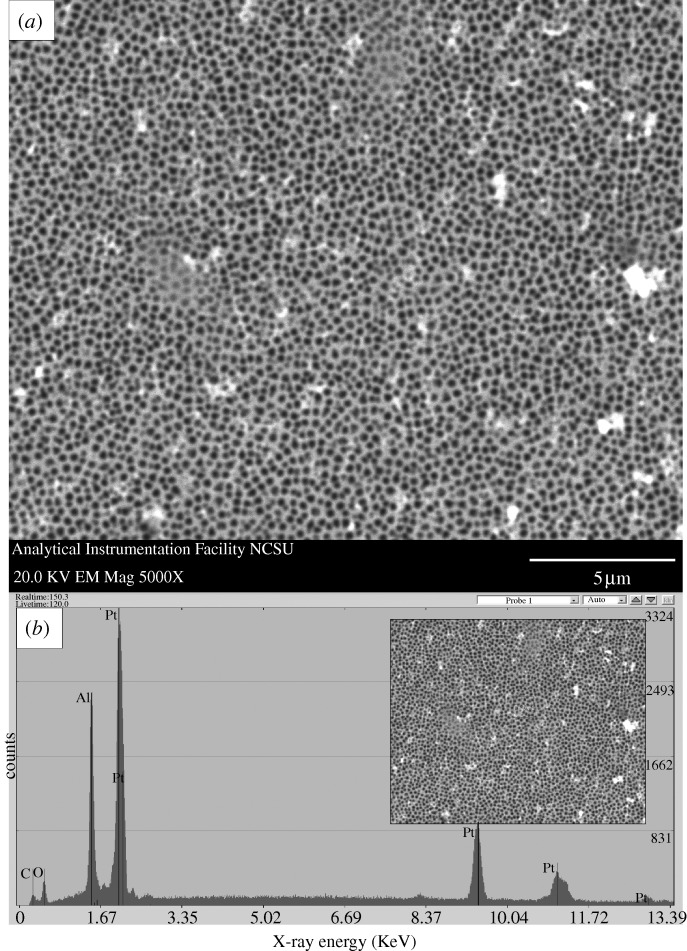
(*a*) Plan-view scanning electron micrograph of a PEGylated, platinum-coated (coating=8 nm) 20 nm pore size nanoporous alumina membrane after treatment with human platelet-rich plasma. (*b*) Energy-dispersive X-ray analysis spectrum for the PEGylated, platinum-coated nanoporous alumina membrane after treatment with human platelet-rich plasma. Protein adsorption, fibrin networks and platelet aggregation were not observed on the surface of the platelet-rich plasma-exposed membrane. The pores largely remain free of fouling. (Reproduced with kind permission from Adiga *et al.* (2008), fig. 2. Copyright 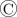 Springer Science+Business Media.)

**Figure 8.**
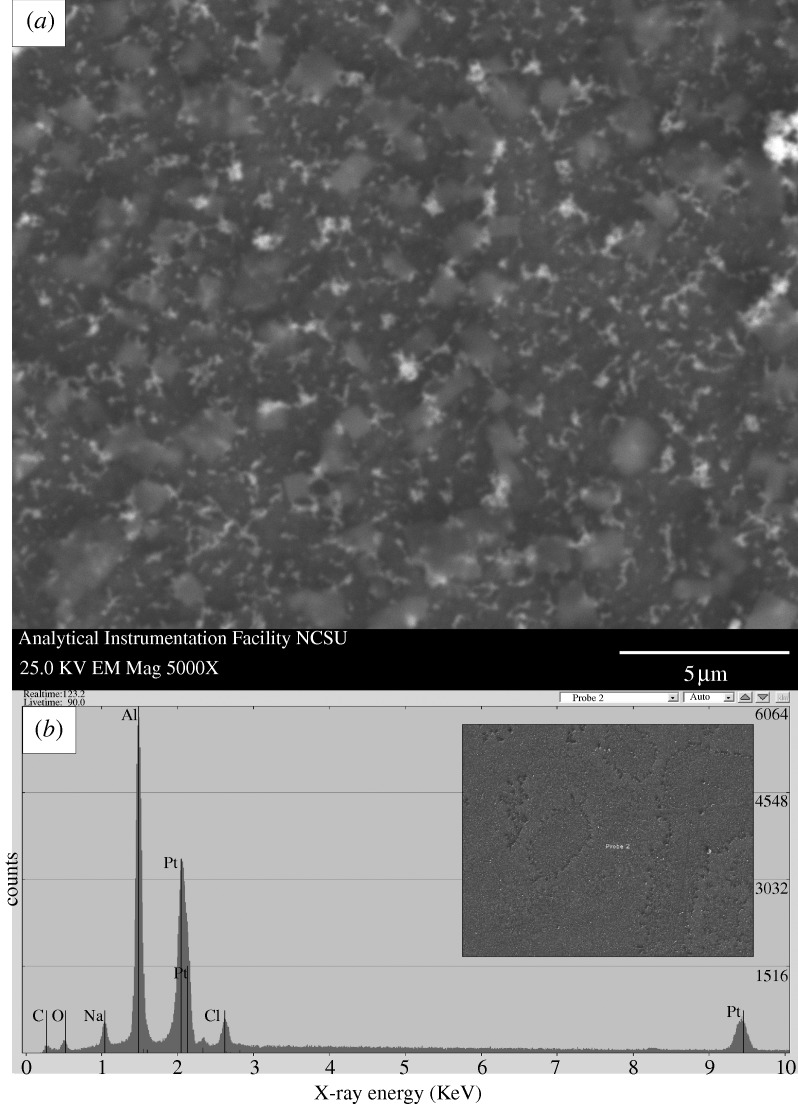
(*a*) Plan-view scanning electron micrograph of a platinum-coated (coating=9 nm) 20 nm pore size nanoporous alumina membrane after treatment with human platelet-rich plasma. (*b*) Energy-dispersive X-ray analysis spectrum for the platinum-coated nanoporous alumina membrane after treatment with human platelet-rich plasma. Protein adsorption and pore fouling were observed on the surface of the platelet-rich plasma-exposed membrane. Sodium chloride crystals were identified on the scanning electron micrograph; sodium and chlorine were noted on the energy-dispersive X-ray spectrum.

**Figure 9.**
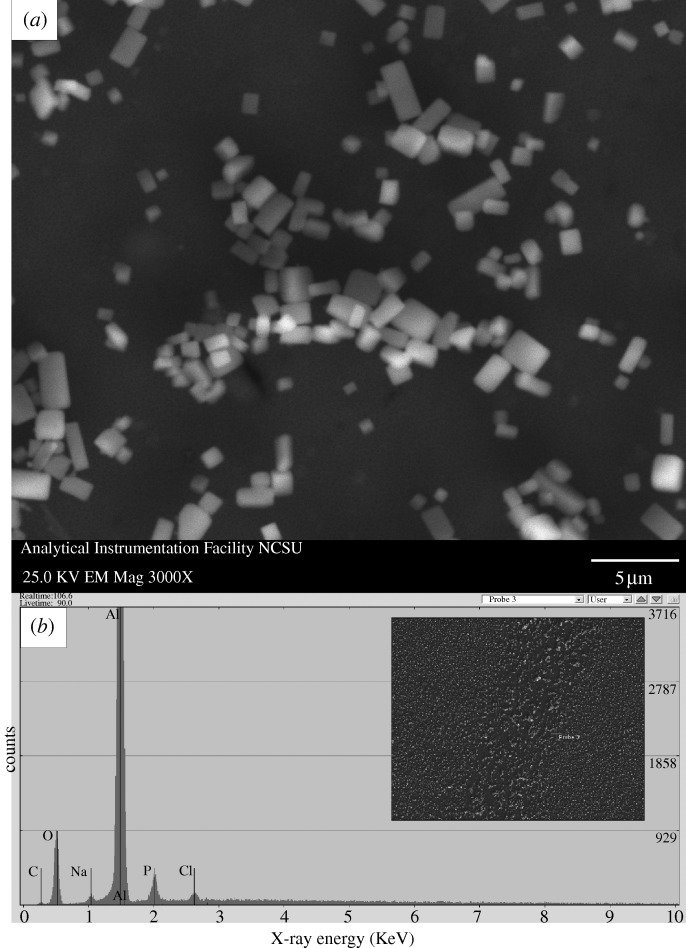
(*a*) Plan-view scanning electron micrograph of an uncoated 20 nm pore size nanoporous alumina membrane after treatment with human platelet-rich plasma. (*b*) Energy-dispersive X-ray analysis spectrum for the uncoated nanoporous alumina membrane after treatment with human platelet-rich plasma. Protein adsorption and pore fouling were observed on the surface of the platelet-rich plasma-exposed membrane. Sodium chloride crystals were identified on the scanning electron micrograph; sodium and chlorine were noted on the energy-dispersive X-ray spectrum.

A scanning electron micrograph of a PEGylated, platinum-coated (coating=8 nm) 20 nm pore size nanoporous alumina membrane after exposure to human platelet-rich plasma is shown in figure 7; no protein aggregation was observed on the surface. The pores of the PEGylated, platinum-coated nanoporous alumina membrane largely remained free of fouling. Scanning electron micrographs of a platinum-coated (coating=9 nm) 20 nm pore size nanoporous alumina membrane and an uncoated 20 nm pore size nanoporous alumina membrane after exposure to human platelet-rich plasma are shown in figures 8 and 9, respectively. Significant protein aggregation and pore fouling were observed on the surfaces of these membranes. In addition, small, widely scattered crystals were observed on the surfaces of the platinum-coated nanoporous alumina membrane and the uncoated nanoporous alumina membrane. The presence of sodium and chlorine in the energy-dispersive X-ray analysis spectra of the platinum-coated nanoporous alumina membrane and the uncoated nanoporous alumina membrane suggests that sodium chloride crystals precipitated from platelet-rich plasma during testing. Recent work by Andara *et al.* (2006) suggested that sodium chloride crystal adsorption is independent from protein adsorption. Tsapikouni & Missirlis (2008) noted that protein adsorption is governed by solution properties (e.g. pH), surface properties (e.g. surface energy) and protein properties (e.g. protein conformation). Previous studies have shown that polyethylene glycol is resistant to adsorption of proteins; for example, Jiang *et al.* (2004) demonstrated that self-assembled monolayers containing oligo(ethyleneglycol)-terminated alkanethiols resist adhesion of mammalian cells as well as non-specific adsorption of proteins; in their study, self-assembled monolayers containing oligo(ethyleneglycol)-terminated alkanethiols on palladium were shown to remain inert for at least four weeks. Seigel *et al.* (1997) utilized an acoustic plate-mode sensor in order to demonstrate that hexa(ethylene glycol)-terminated self-assembled monolayers demonstrate very low protein adsorption. Wang *et al.* (1997) suggested that oligo(ethylene glycol)-terminated self-assembled monolayers resist proteins and other biological molecules owing to the fact that these molecules form hydrogen bonds with water molecules. It is believed that a stable interfacial layer of water molecules inhibits protein–surface contact and prevents protein adsorption; as such, this process is considered to be enthalpic instead of entropic. These antifouling properties are appealing for implantable devices, since protein fouling and cell adhesion can impede transport of biological molecules between the device and the surrounding tissues.

Nanoporous materials of 20 nm pore size with resistance to fouling by human platelet-rich plasma have potential uses in implantable biosensors. According to Tsapikouni & Missirlis (2008), proteins are adsorbed to the surface of biomaterials within seconds after implantation within the body. For example, Hoshino *et al.* (2009) noted that the primary factor that is limiting development of a mechanical artificial pancreas is the absence of an *in vivo* glucose sensor that can function within the body for extended periods of time. Vriesendorp *et al.* (2005) showed that currently used continuous glucose-monitoring devices that contain subcutaneous glucose sensors possess insufficient accuracy for use in perioperative medical care. Membranes are typically used to protect the functional component (e.g. electrode) of the biosensor from biofouling as well as to provide a diffusion barrier that enables transport of the analyte and prevents transport of other species. Wisniewski & Reichert (2000) suggested that biosensor failure is commonly attributed to membrane biofouling; adhesion or adsorption of small proteins, large proteins as well as cells may occur on a biosensor membrane, limiting the transport of analytes from the surrounding tissues to the sensor. For example, Kerner *et al.* (1993) demonstrated a decrease in implantable sensor sensitivity after placement in human plasma as well as in human plasma ultrafiltrate; this loss of sensor sensitivity was shown to be reversible upon exposure of the sensor to a buffer solution. Studies by Mǎdǎraş & Buck (1996) and others have indicated that biosensor membranes should exhibit low protein fouling, controlled porosity (e.g. minimal pore-to-pore variation) as well as low thickness values in order to enable the biosensor to rapidly respond to fluctuations in analyte concentrations. Uehara *et al.* (2009) suggested that nanoporous membranes could be incorporated within implantable biosensors for monitoring of blood glucose levels over extended periods of time; nanoporous membranes prepared using polyethylene-block-polystyrene were shown to enable diffusion of glucose but prevent diffusion of albumin. However, the cell or blood compatibility of this device was not described. In addition, Desai *et al.* (2000) noted that hydrophobic or hydrophilic groups in polymeric sensor membranes can promote nucleation of calcium phosphate, which can interfere with membrane performance. Several investigators have examined the use of porous silicon as a biosensor material. For example, Letant *et al.* (2003) demonstrated that functionalized silicon membranes were able to capture streptavidin-coated beads that served as simulated bio-organisms. Desai *et al.* (2000) demonstrated that micromachined silicon membranes allowed glucose diffusion and prevented albumin diffusion. It should be noted that porous silicon may undergo degradation under physiological conditions; for example, Canham *et al.* (1997) noted that porous silicon undergoes calcification in acellular simulated body fluids. In addition, Anderson *et al.* (2003) showed that porous silicon films degrade in aqueous solutions; significant release of Si(OH)_4_ was noted at a temperature of 37^°^C and at pH values of 7 and higher. Materials with greater porosity demonstrated higher dissolution rates. Finally, Foell *et al.* (2002) noted that it is, at present, difficult to obtain self-ordered pores in porous silicon.

**Figure 10.**
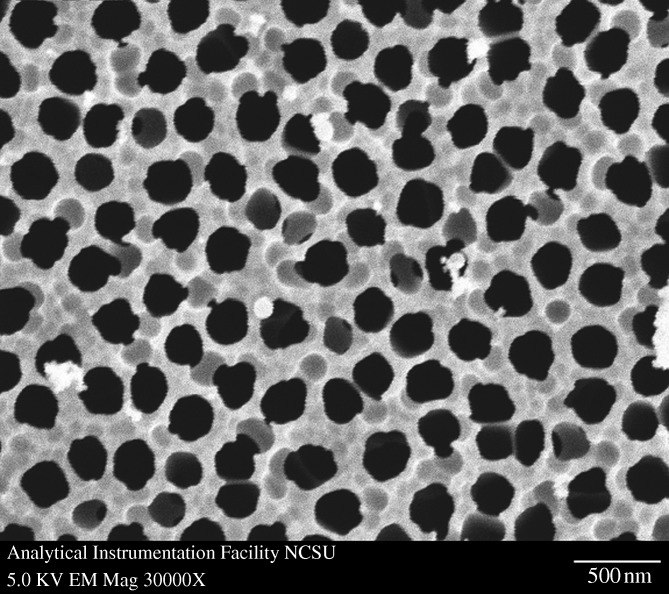
Plan-view scanning electron micrograph of a zinc oxide-coated (coating= 5 nm) 100 nm pore size nanoporous alumina membrane.

**Figure 11.**
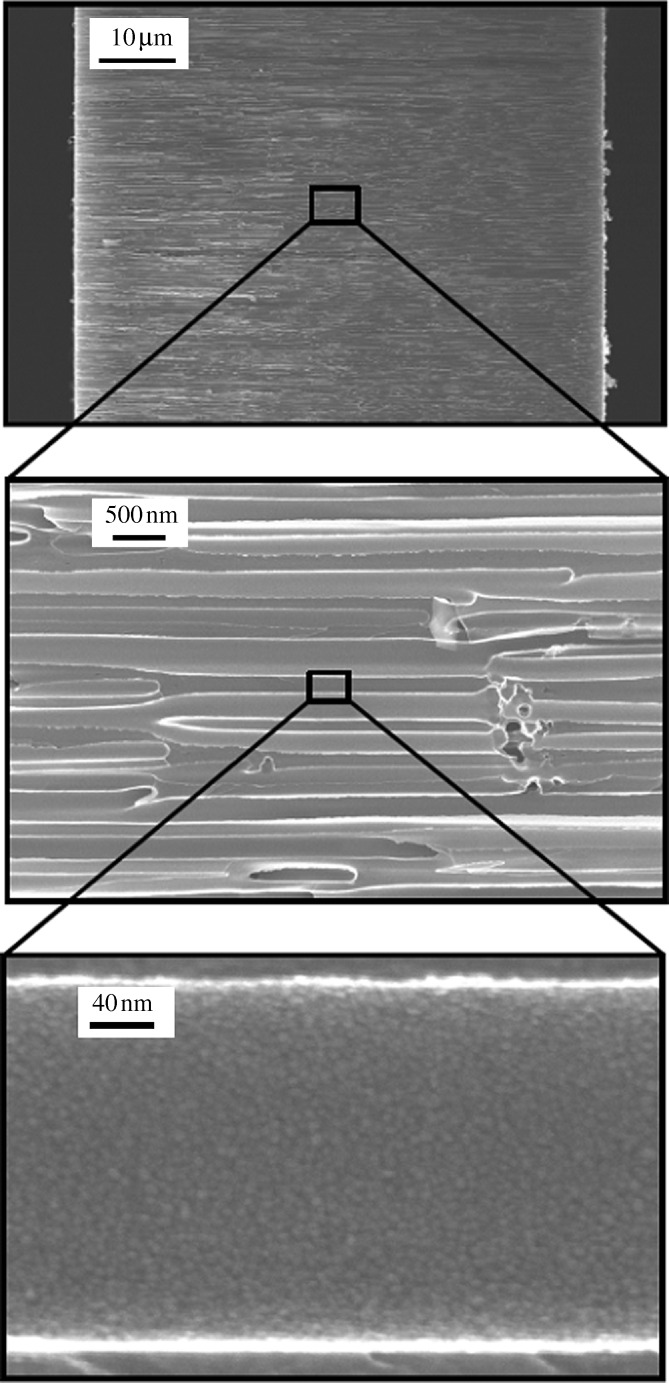
Cross-sectional scanning electron micrographs obtained from a cleaved specimen of a nanoporous alumina membrane following atomic layer deposition of a 5 nm zinc oxide coating.

**Figure 12.**
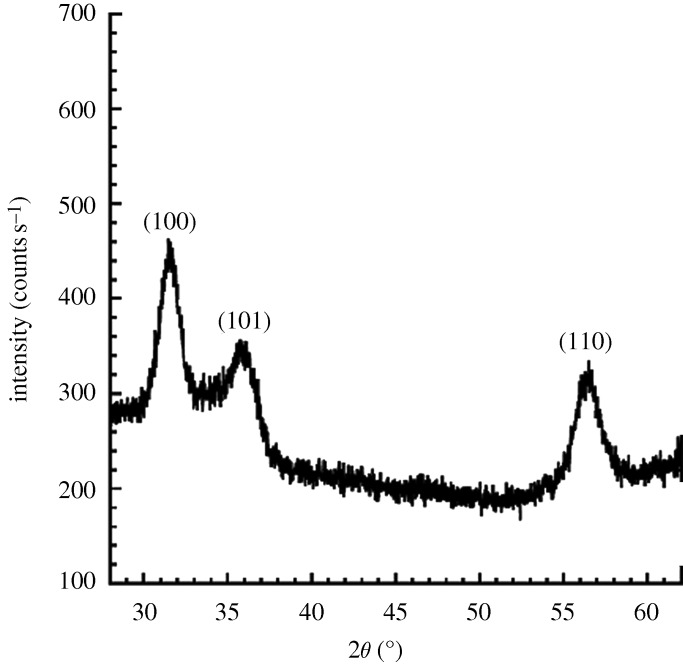
X-ray diffraction pattern for a zinc oxide-coated (coating= 5 nm) nanoporous alumina membrane, which contains peaks that correspond to hexagonal zincite.

**Figure 13.**
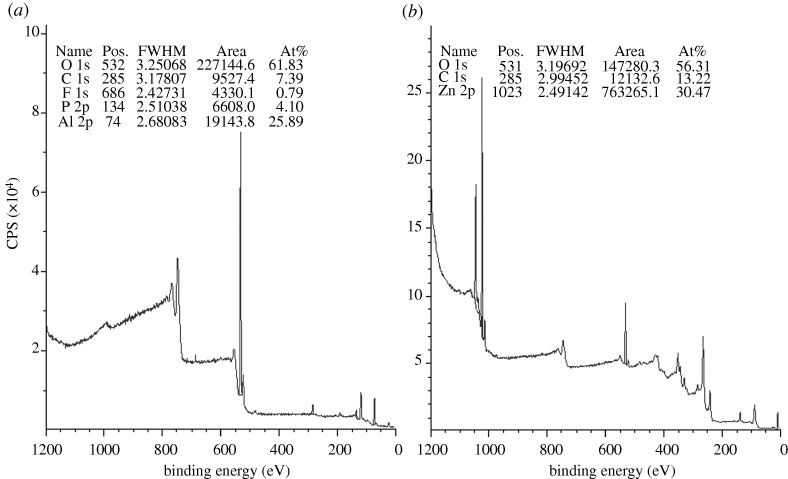
(*a*) X-ray photoelectron spectrum of an uncoated 100 nm pore size nanoporous alumina membrane. (*b*) X-ray photoelectron spectrum of a zinc oxide-coated (coating=5 nm) 100 nm pore size nanoporous alumina membrane.

Figure 10 shows a plan-view scanning electron micrograph of a 100 nm pore size nanoporous alumina membrane following atomic layer deposition of a 5 nm zinc oxide coating. This figure demonstrates that the nanoporous alumina membrane coated with zinc oxide using atomic layer deposition exhibited monodisperse pore sizes and high porosity. Figure 11 shows cross-sectional scanning electron micrographs obtained from a cleaved nanoporous alumina membrane following atomic layer deposition of a 5 nm thick zinc oxide coating. Figure 11*b* indicates that the alumina pore structure is not completely uniform; branching of pores as well as intersections of pores are shown. Figure 11*c* shows that the inner surfaces of the pores are completely coated with zinc oxide nanocrystals that exhibit dimensions between 5 and 10 nm. In addition, cross-sectional energy-dispersive X-ray analysis was performed across the membrane thickness; the zinc oxide concentration was shown to be uniform throughout the membrane. Figure 12 shows an X-ray diffraction pattern for a nanoporous alumina membrane following atomic layer deposition of a 5 nm zinc oxide coating. The peaks in this figure match the expected positions for hexagonal zincite, which are described in File 36-1451 of the Joint Committee on Powder Diffraction Standards (2000). The size of the zinc oxide crystals was estimated from peak widths using the Scherrer formula to be 8 nm. Uncoated nanoporous alumina membranes are composed of amorphous alumina and impurities; X-ray diffraction measurements performed on uncoated nanoporous alumina membranes yielded no peaks. Figure 13*a* contains an X-ray photoelectron spectrum of an uncoated 100 nm pore size nanoporous alumina membrane. Figure 13*b* contains an X-ray photoelectron spectrum of a zinc oxide-coated (coating=5 nm) 100 nm pore size nanoporous alumina membrane. The characteristic 2p peak for aluminium (binding energy=74 eV) was not detected in the spectrum of the zinc oxide-coated nanoporous alumina membrane. Analysis using CasaXPS software (RBD Instruments, Bend, USA) confirmed the absence of aluminium; the zinc oxide coating was shown to be continuous and was shown to completely cover the nanoporous alumina membrane surface.

**Figure 14.**
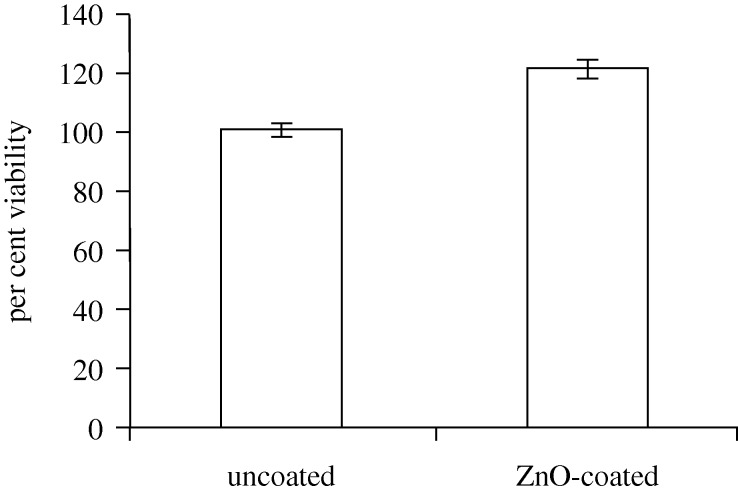
The 24 h MTT viability assay data for the uncoated 100 nm pore size nanoporous alumina membrane and the zinc oxide-coated (coating=5 nm) 100 nm pore size nanoporous alumina membrane. Data were standardized by the uncoated membrane control. The zinc oxide-coated membranes demonstrated higher viability than the uncoated membrane.

The 24 h MTT viability assay data for the uncoated 100 nm pore size nanoporous alumina membrane and the zinc oxide-coated (coating=5 nm) 100 nm pore size nanoporous alumina membrane are shown in figure 14. The data for the coated membrane were standardized by the data for the uncoated membrane. The zinc oxide-coated nanoporous alumina membranes were shown to support higher cell viability than the uncoated nanoporous alumina membranes. It should be noted that Naji & Harmand (1991) previously used *in vitro* assays to confirm the cytocompatibility of amorphous alumina. The increase in cell proliferation may be attributed to cell interactions with released zinc ions. MacDonald (2000) indicated that Zn^2+^ activates mitogen-activated protein kinase, which is involved with cell proliferation. Chen *et al.* (1999) showed that zinc directly promotes DNA synthesis; zinc was shown to increase cell DNA content. Hershfinkel *et al.* (2001) demonstrated that human epidermal keratinocytes possess a functional extracellular zinc-sensing receptor; their work suggests that the detection of extracellular zinc results in activation of numerous signal transduction pathways and enhanced keratinocyte proliferation. Work by MacDonald (2000), McNeil *et al.* (1998) and Oda *et al.* (2000) also suggested that keratinocyte proliferation may be enhanced by zinc.

**Figure 15.**
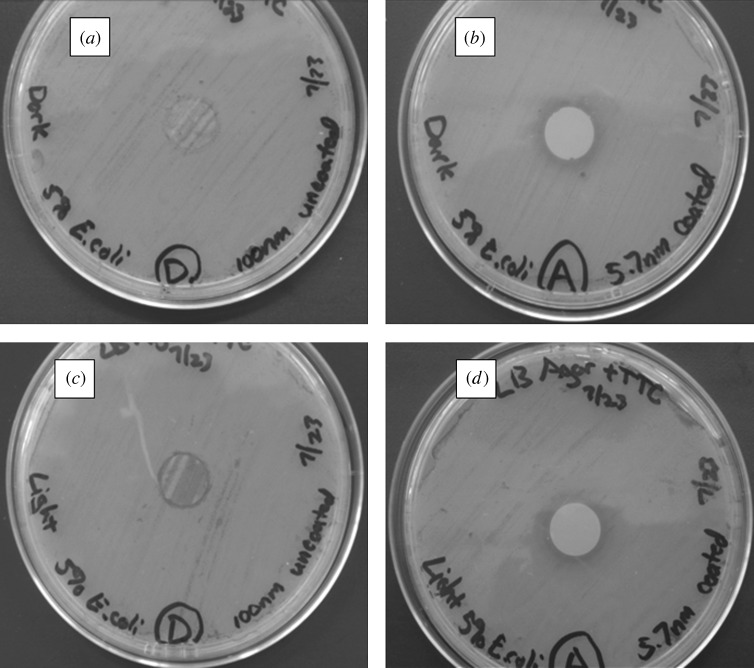
Light microscopy images of agar plating assay results after 24 h of incubation. Materials were examined on Luria–Bertani agar plates, which were inoculated with *E. coli*. (*a*) Uncoated 100 nm pore size nanoporous alumina membrane without light exposure. (*b*) Zinc oxide-coated (coating= 5 nm) 100 nm pore size nanoporous alumina membrane without light exposure. (*c*) Uncoated 100 nm pore size nanoporous alumina membrane under continuous light exposure. (*d*) Zinc oxide-coated (coating= 5 nm) 100 nm pore size nanoporous alumina membrane under continuous light exposure.

**Figure 16.**
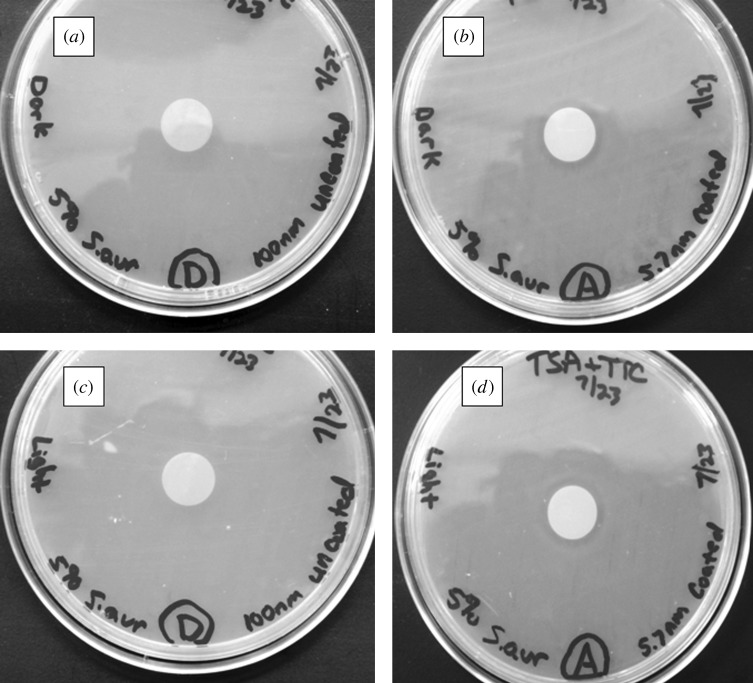
Light microscopy images of agar plating assay results after 24 h of incubation. Materials were examined on tryptic soy agar plates, which were inoculated with *S. aureus*. (*a*) Uncoated 100 nm pore size nanoporous alumina membrane without light exposure. (*b*) Zinc oxide-coated (coating= 5 nm) 100 nm pore size nanoporous alumina membrane without light exposure. (*c*) Uncoated 100 nm pore size nanoporous alumina membrane under continuous light exposure. (*d*) Zinc oxide-coated (coating= 5 nm) 100 nm pore size nanoporous alumina membrane under continuous light exposure.

Agar plating assay results for the zinc oxide-coated 100 nm pore size nanoporous alumina membranes and uncoated nanoporous alumina membranes are shown in figures 15 and 16. Figure 15 shows light microscopy images of agar plating assay results after 24 h of incubation for uncoated and coated membranes, which were examined on Luria–Bertani agar plates inoculated with *E. coli*. Figure 16 shows light microscopy images of agar plating assay results after 24 h of incubation for uncoated and coated membranes, which were examined on tryptic soy agar plates inoculated with *S. aureus*. The uncoated nanoporous alumina membranes showed no inhibition of growth of *E. coli* and *S. aureus* under either continuous light or dark exposure. On the other hand, zinc oxide-coated nanoporous alumina membranes inhibited the growth of *E. coli* and *S. aureus* on the membrane surface. A small zone of growth inhibition was observed around each zinc oxide-coated membrane, indicating leaching of zinc oxide from the membrane. There was no discernable difference in antimicrobial performance between membranes examined under either continuous light or dark exposure.

Zinc oxide exhibits several unusual physical and biological characteristics that make it an appropriate selection as an antimicrobial coating material. As Sawai (2003) noted, zinc oxide is more stable at high pressures and high temperatures than conventional organic antimicrobial pharmacological agents. In addition, Kamat *et al.* (2002) demonstrated that zinc oxide coatings can be used for simultaneous sensing and degradation of organic contaminants, since the presence of a contaminant may be detected by quenching of visible emission from zinc oxide. According to Li *et al.* (2008), the broad level of antimicrobial activity provided by zinc oxide remains unclear; however, several possible mechanisms of activity have been presented in the literature. Akhavan *et al.* (2009) have shown that zinc oxide provides antimicrobial activity in both dark and lighted experimental conditions. Sawai *et al.* (1995) demonstrated that zinc oxide exhibits antimicrobial activity against both Gram-positive and Gram-negative bacteria. A study by Atmaca *et al.* (1998) demonstrated activity by zinc against *S. aureus*, *Staphylococcus epidermidis* and *Pseudomonas aeruginosa* bacteria; this study suggested that zinc interacts with microbial membranes and acts to prolong the lag phase of the growth cycle. Liu *et al.* (2009) recently suggested that zinc oxide distorts and damages the bacterial cell membrane; this process results in leakage of intracellular contents and cell death. Huang *et al.* (2008) also demonstrated disorganization of Gram-negative membranes and Gram-positive membranes after interaction with zinc oxide. Work by Zhang *et al.* (2007) indicated that zinc oxide causes damage to the membrane wall of *E. coli*; their work suggested that zinc oxide acts either by means of reactive oxygen species (e.g. hydrogen peroxide) generation or by means of zinc oxide–membrane wall interaction. In addition, Sawai *et al.* (1998) indicated that zinc oxide releases hydrogen peroxide, which crosses the cell membrane and causes intracellular damage. It should be noted that degradation of the entire zinc oxide coating could potentially result in a loss of antimicrobial activity; as such, optimized zinc oxide-coated membranes should exhibit low rates of zinc oxide release. However, work by Yebra *et al.* (2006) noted that zinc oxide-based paints exposed to saline (sea water) solutions exhibited markedly lower degradation rates than conventional cuprous oxide-based paints. In addition, Pilbath *et al.* (2008) have demonstrated that 1,5-diphosphonic acid-based treatments may be used to inhibit degradation of zinc surfaces.

Nanoporous materials of 100 nm pore size with antimicrobial activity have potential uses in water purification (Savage & Diallo 2005; Smith 2006). Marcucci *et al.* (2003) have indicated that the use of membranes for water purification is growing. Owing to their thermal stability, nanoporous ceramic filters can be cleaned using high-temperature procedures (e.g. autoclaving) for repeated use. The Gram-negative and Gram-positive organisms that were examined in this study, *E. coli* and *S. aureus*, are organisms that were noted by Lechevallier & Seidler (1980) as well as Edberg *et al.* (2000) to be found in drinking water; these pathogenic micro-organisms have been shown to cause human illness. Van der Bruggen & Vandecasteele (2003) have described the use of nanoporous membranes for removing particles as well as micro-organisms. Ma, B. *et al.* (2009) and Ma, N. *et al.* (2009) noted that membranes have several benefits over conventional processes for removal of micro-organisms and particles during water treatment processes; membrane-based processes involve lower energy costs, more compact devices, more scalable processes and more straightforward processes than conventional processes. Peltier *et al.* (2003) demonstrated that nanofiltration may improve water quality; significant reductions in biological and organic contaminants were achieved. For example, Srivastava *et al.* (2004) described the use of carbon nanotube filters as water purification membranes; these hollow cylinders with aligned carbon nanotube walls were shown to remove *E. coli*, *S. aureus* and *Poliovirus sabin 1*. However, Liu *et al.* (2010) have stated that microbial biofouling of membranes is the leading factor that limits the use of nanoporous membranes in water treatment. Biofouling involves microbial adhesion and proliferation on the water purification membrane surface; according to Hilal *et al.* (2004), bacteria may form confluent biofilms on the membrane surface. Kochkodan *et al.* (2008) and Liu *et al.* (2010) noted that micro-organisms degrade membrane permeability, thereby reducing membrane lifespan and increasing the amount of energy required for filtration. In addition, Park *et al.* (2005) suggested that micro-organisms may release by-products that degrade water quality. Flemming *et al.* (1997) noted that the conventional mechanism for preventing membrane biofouling, pre-treatment of water with a biocidal agent, may not be completely effective owing to rapid proliferation of many micro-organisms; according to Kim *et al.* (2009), biocidal agents may damage the membrane surface. In addition, Krasner *et al.* (2006) showed that conventional disinfectants such as free chlorine, chloramines and ozone may interact with constituents of treated water to create carcinogenic disinfection by-products. Li *et al.* (2008) stated that membrane biofouling may be minimized by placing biocidal agents on the membrane surface; the antimicrobial membrane surface may serve to prevent microbial adhesion and proliferation as well as to provide separation functionality. Haas (2000) and Weber (2002) suggested that nanoporous membranes with antimicrobial properties may be especially useful small-scale or point-of-use systems; distributed water systems may be especially useful for underdeveloped regions, remote locations and emergency situations. Nanoporous membranes containing chlorine-free biocidal agents may provide an environmentally friendly mechanism for treating waterborne micro-organisms. Several investigators have recently evaluated the use of inorganic membranes containing the photoactive anatase phase of titania for water filtration and photocatalysis. For example, Ma, N. *et al.* (2009) deposited an Si-doped TiO_2_ photocatalytic layer on an alumina membrane using a sol–gel technique. Water permeability was shown to be related to the thickness of the titania layer. In addition, photocatalysis of a dye under ultraviolet irradiation was demonstrated. Zhang *et al.* (2006) prepared silica/titania nanotube composite membranes on porous alumina using a sol–gel method; removal of Direct Black 168 dye by means of membrane separation and photocatalysis was demonstrated. Zhang *et al.* (2008) grafted anatase titania nanotubes within the channels of alumina membranes using a liquid-phase deposition mechanism; these membranes demonstrated photodegradation of humic acid. In addition, the membranes demonstrated the removal of humic acid from water. It should be noted that titania-containing nanoporous membranes rely on access to a light source for photocatalytic antimicrobial activity; incorporation of a light source within the water treatment environment may require extensive retrofitting as well as additional cost of operation. In addition, Li *et al.* (2008) suggested that a light-dependent photocatalytic antimicrobial system may have a lower disinfection efficiency than a conventional slurry reactor.

## 4. Conclusions

The results of this study indicate that atomic layer deposition may be used to modify the pores of nanoporous alumina membranes for medical and environmental health applications. The pores of the PEGylated platinum-coated nanoporous alumina membranes remained free of fouling after exposure to human platelet-rich plasma; protein adsorption, fibrin networks and platelet aggregation were not observed on the membrane surface. Zinc oxide-coated nanoporous alumina membranes demonstrated activity against two waterborne pathogens, *E. coli* and *S. aureus*. Atomic layer deposition is a scalable, cost-effective method for modifying the surfaces of materials; it is anticipated that this technique can successfully compete on a commercial basis with conventional surface modification processes. The development of novel nanoporous membranes with anti-protein biofouling and antimicrobial functionalities could have significant impacts on *in vivo* sensing and water purification, respectively. Future studies will involve assessing the size selectivity of coated nanoporous alumina membranes. For example, efforts are needed to incorporate ‘smart’ functionalities such as biomimetic selective transport properties within the pores of nanoporous alumina membranes (Adiga *et al.* 2008)
